# Intestinal transplantation in Familial Adenomatous Polyposis

**DOI:** 10.1007/s10689-025-00468-6

**Published:** 2025-05-03

**Authors:** Emilio Canovai, Sarah Upponi, Irum Amin

**Affiliations:** 1 Cambridge Centre for Intestinal Rehabilitation and Transplant (CamCIRT), Cambridge, UK; 2 Oxford Transplant Centre, Churchill Hospital, Oxford University Hospitals, Headington, UK; 3 Department of Radiology, Addenbrooke’s Hospital, Cambridge, UK; 4 Department of Transplant Surgery,, Addenbrooke’s Hospital, Cambridge, UK

**Keywords:** Intestinal transplantation, Desmoids, Familial adenomatous polyposis

## Abstract

In patients with Familial Adenomatous Polyposis (FAP), large desmoid tumors can develop all over the body. However, the most frequent presentation is as large intra-abdominal masses, usually located in the mesentery of the small bowel. From there, they tend to grow and invade both the abdominal wall and/or the retroperitoneal structures. This can cause life-threatening complications such as recurrent abdominal sepsis with fistulation and damage to vital organs. In selected patients, the only option may be radical resection and replacement by intestinal transplantation (ITx). We aimed to review all the current literature on ITx for FAP-related desmoids and provide an update from the largest single-center experience (2007–2024). All patients undergoing ITx for FAP-related desmoid were included. Between 2007 and 2024, 166 ITx was performed in 158 patients at Addenbrooke’s Hospital, Cambridge, UK. Of these, 20 (12%) were for desmoid associated with FAP (10 modified multivisceral transplants, 8 isolated ITx and 2 liver-containing grafts). The five-year all-cause patient survival was 92%, median follow-up was 4.3 years. As the patients presented with very advanced disease, many technical challenges were faced such as: extensive ureteric involvement, abdominal wall fistulation, management of previously formed ileo-anal pouches and extra-abdominal recurrences. Graft selection was another evolving issue, as foregut resection- versus sparing techniques require careful preoperative risk stratification due to increased long-term cancer risk in FAP patients. For certain patients with advanced FAP/desmoid disease, ITx can allow for a radical resection with excellent survival and functional outcomes. However, there is a high degree of initial morbidity associated with the operation and patients should be appropriately counselled. Graft selection and degree of native organ resection requires a careful balanced discussion.

## Introduction

Familial Adenomatous Polyposis (FAP) is a hereditary cancer syndrome that results in multiple adenomatous polyp formation which significantly increases the risk of colorectal cancers and desmoid tumor formation [1].

With the advent of screening and prophylactic colectomies, there has been a significant decline in the risk of death by colorectal cancer [2]. Instead, patients can be faced with two challenging problems later in their life: complex desmoid disease and upper GI tract adenomas/malignancies.

Desmoids are fibroblast tumors that can occur anywhere, but in FAP patients they tend to be located intra-abdominally. The natural history is difficult to predict and can range from regression, stable in size or sudden rapid growth. In the latter case, this can lead to central necrosis, abscess formation and fistulation [3].

What makes intra-abdominal desmoid tumors especially challenging is that they tend to develop within the small bowel mesentery [4]. As these grow, tumors can eventually cause intestinal obstruction and intestinal failure. Due to proliferation in the mesentery, surgical resections are avoided if possible due to the high recurrence rate and potential loss of significant amounts of small bowel. Therefore, the initial focus is on non-surgical treatments such as radiation therapy or systemic treatments such as NSAIDS, chemotherapy, hormonal therapy and tyrosine kinase inhibitors [5].

However, in those patients where the disease continues to progress, surgical resection becomes unavoidable. This is mainly in cases where desmoids become very large and compression lead to significant pain, recurrent abscess formation and/or invasion of surrounding structures. Conventional resection surgery is very challenging due adhesions, proximity to vital organs and very high recurrence rates despite complete resection margins [6].

The other main challenge in FAP patients, is the high risk of upper GI (stomach or duodenal) adenocarcinomas which is one of the leading disease-related causes of death in FAP [7, 8]. This requires careful endoscopic monitoring but this this may become very difficult in those patients who develop numerous polyps with ‘carpeting polyps’ in large sections of the upper GI tract [9]. These patients may require a gastrectomy and/or duodenectomy if screening is no longer viable and the malignancy risk becomes too high. The most frequent risk stratification system for duodenal malignancy is the Spigelman classification which combined polyp number and size with histological features (morphology and dysplasia rates) [10].

In certain cases, the desmoid disease is so extensive that Intestinal Transplantation (ITx) may be considered (Table 1). Several centers, including our own, have reported on experience of combining a complete intestinal resection and subsequent ITx [11–17]. This allows for intestinal function to be maintained while removing all of the desmoid disease.

The downside is that this adds all the known risks of transplantation such as chronic immunosuppression, rejections, infections, malignancies and graft-versus-host disease. Furthermore, the surrounding structures, especially the retroperitoneal ones, will require meticulous exploration and preservation. A final important question relates to which native organs require resection and which graft should be selected for each patient.

In this study, we aim to provide an overview of the indications, challenges and outcomes of transplantation in FAP patients with extensive desmoid disease.

**Table 1:** Indication for intestinal transplantation

Irreversible intestinal failure, plus
1 Life-threatening complications of parenteral nutrition
b) Severe sepsis
• More than one life-threatening episode of catheter-related sepsis for which no remediable cause can be identified by a recognised intestinal failure centre
c) Limited central venous access
• Venous access limited to three major conventional sites in adults (above and below the diaphragm) and two major conventional sites above the diaphragm in children
• Conventional central venous sites are defined as internal jugular, subclavian and femoral veins
1.1.2. Very poor quality of life thought likely to be correctable by transplantation
1.2. Patients with indications for extensive surgery involving partial or complete evisceration
1.2.1. Surgery to remove a large proportion of the abdominal viscera which is considered untenable without associated multi-visceral transplantation/isolated small bowel transplantation (e.g. extensive desmoid disease, extensive severe mesenteric arterial disease requiring intervention)
1.2.2. Localised malignancy considered amenable to curative resection which would necessitate extensive evisceration (e.g. localised neuroendocrine tumours).
1.3. Patients requiring transplantation of other organs where exclusion of simultaneous intestinal transplantation would adversely affect patient survival
1. Where the transplantation procedure is expected to preclude the possibility of future intestinal transplantation (e.g. loss of venous access or further human leukocyte antigen sensitisation)
2. Where the need for subsequent intestinal transplantation is considered likely and the risk of death is increased by excluding the intestine from the graft
Examples include predictable problems related to administering immunosuppression (e.g. line sepsis), or continuing severe intestinal disease such as diabetic visceral neuropathy, or ultra-short bowel syndrome, which may cause fluid, electrolyte and acid base balance problems that would damage an existing or planned renal graft
1.4 Inclusion of a renal graft at the time of intestinal or multi-visceral transplantation
It is recommended that adults with corrected GFR of < 45 mls/min/m2 are evaluated for the possibility of simultaneous renal transplantation.

## Methods

For this study we reviewed all published literature on ITx for desmoids. Second, we updated the date on our previously published series [11].

We reviewed our database between the start of the program in 2007 up to 2024. We included all patients receiving an ITx graft for FAP-related desmoid disease. Non-FAP desmoids were excluded from this series. Most of our referrals originated from the UK’s national FAP unit: St Mark’s Centre for Familial Intestinal Cancer in London.

The study analyzed basic patient demographics such as age, weight, gender, presence of intestinal failure, use of parenteral nutrition, prior medical treatments, rejection rate and treatments, graft- and patient survival. The extent of desmoid disease at time of ITx was categorized according to the Church system [18]. Prior surgical resections, amount of native GI tract left at time of ITx and presence of a J-pouch were also recorded.

Our immunosuppression protocol was based on Alemtuzumab and methylprednisolone induction, followed by tacrolimus (initial trough levels of 8–12 ng/ml), mycophenolate mofetil (500 mg per day) and maintenance prednisolone [19].

ITx graft types were labelled as **isolated (**isolated ITx**)** (duodenum, pancreas, small bowel – and partial colon), **multivisceral (MVT)** (stomach, duodenum, pancreas, small bowel and partial colon) and **modified multivisceral** (**modified MVT**) [20].

Ethics declaration: At the time of their transplantation listing, all patients provided informed consent for the anonymous use of their data in retrospective research.

## Results

In the period 2007 to 2024, we performed 20 ITx for FAP-induced desmoid disease (see Table 2). This accounted for 12% of our total ITx volume (158 patients receiving 166 transplants). Most of the patients either had advanced desmoid tumors or had intestinal failure complications after significant resections. As could be expected in this population, most had prophylactic pan-proctocolectomies and 5 had a J-pouch at the time of their transplantation.

### Ongoing abdominal sepsis and loss of abdominal domain

**Fig. 1:**
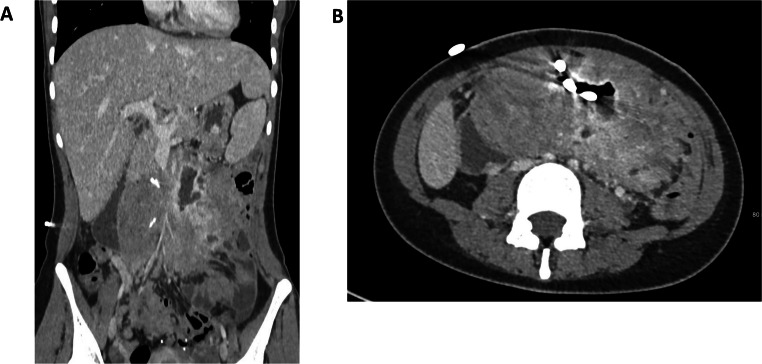
CT A coronal and B axial images – diffuse mesenteric desmoid with small bowel fistulas and central cavity with drain in situ

Ten patients had complex desmoid-induced fistulating disease with either enterocutaneous or entero-enteric fistulae. These patients often had extensive – Church grade 4 desmoid disease. Many presented with refractory abdominal collections and were dependent on drains and/or multiple courses of antibiotics (see Fig. 1).

**Table 2:** Patient date before and during transplantation

Patient number	Age	Gender	Indication for transplant	Underlying APC mutation	Type of graft	Previous other surgeries	Ureteric involvement	Extra-abdominal involvement	On parenteral nutrition at time of ITx
1	37	F	Extensive desmoid disease requiring resection due to vascular compromise, large growing duodenal polyp	Not available	MVT	No	**None**	None	N
2	35	F	Extensive desmoid disease requiring resection due to ureteric compromise	codon 561	Modified MVT + kidney	High defunctioning jejunostomy due to recurrence	**Y - left ureter (nephrectomy)**	None	Y
3	28	M	IFALD due to SBS after resection	Not available	Modified MVT	Subtotal Enterectomy due to mesenteric recurrence	**None**	None	Y
4	44	F	Ultrashort bowel after extensive resection	codon 1450	Isolated ITx	Subtotal Enterectomy due to mesenteric recurrence	**None**	Chest wall	Y
5	34	F	Extensive desmoid disease requiring resection due to symptoms, left ultrashort	codon 1491	Isolated ITx	Subtotal enterectomy due to mesenteric recurrence	**None**	None	Y
6	38	F	IFALD + Extensive desmoid disease requiring resection, ultrashort	Not available	MVT	Subtotal enterectomy due to mesenteric recurrence	**None**	None	Y
7	34	F	IFALD + extensive desmoid with abdominal wall compromise and ureteric obstruction	Not available	Isolated ITx + renal auto-transplant	partial gastrectomy and roux-en-y reconstruction	**Y - right auto transplant**	Chest and abdominal wall	Y
8	34	M	Fistulating desmoid disease with ureteric obstruction	codon 1309	Modified MVT + renal auto-transplant	Previous J pouch reconstruction	**Y - right auto transplant**	Abdominal wall	N
9	37	M	Fistulating desmoid disease	codon 564	Modified MVT	Previous J pouch, Abscess drainage, laparostomy	**None**	None	Y
10	52	F	IFALD due to SBS after resection		Isolated ITx	Subtotal Enterectomy	**None**	None	Y
11	30	F	Extensive fistulating desmoid disease requiring resection with ureteric compromise	codon 1068	Modified MVT	Previous J pouch, Drain placement due to fistulating disease	**Y - encasing the left ureter**	None	N
12	34	M	IFALD due to SBS after resection	Not available	Isolated ITx	subtotal enterectomy	**None**	None	Y
13	35	F	Fistulating desmoid disease – abdominal wall compromised	codon 1465	Modified MVT	No	**None**	None	Y
14	35	M	Extensive desmoid disease requiring resection – abdominal wall compromised		Isolated ITx	No	**None**	None	N
15	50	M	Extensive desmoid disease requiring resection – abdominal wall compromised	del exons 1–15	Modified MVT	No	**None**	None	N
16	20	F	Fistulating desmoid disease	codon 1122	Modified MVT	No	**Y – left ureter**	None	Y
17	45	M	desmoid invading mesentery, intestinal failure	Not available	Modified MVT	No	**Y – bilateral encasement**	None	Y
18	33	M	Desmoids, panproctocolectomy, SBS and complex fistulae	Not available	Isolated ITx	No	**None**	None	Y
19	51	M	Desmoids causing ureteric obstruction/sepsis, intestinal failure and obstruction	Not available	Isolated ITx + kidney	Pancreaticoduodenectomy due to duodenal adenocarcinoma	**Y- native horseshoe kidney encasement**	None	Y
20	58	F	SBS, loss of vascular access, ureteric obstruction	codon 554	Modified MVT	Subtotal enterectomy	**Y- unilateral ureteric encasement**	None	Y

**Legend**: APC = adenomatous polyposis coli, **IFALD** = intestinal failure associated liver disease, **SBS =** short bowel syndrome, **ITx**; Intestinal Transplant, **MVT**; Multivisceral Transplant

The disease frequently impacted the abdominal wall which was either directly invaded by the desmoid disease or retracted with loss of domain due to previous intestinal resections. To aid with abdominal closure, 12 patients required advanced techniques. The non-vascularized rectus fascia was used in the majority of these cases (*n* = 10).

### Ileo-anal pouch issues

As common in the FAP population, most had previously undergone prophylactic colectomies. In addition, 5 patients in this cohort had ileo-anal J-pouches. As the desmoid disease frequently involved the mesentery traveling down to the pouch, a pelvic resection at time of ITx would be very complicated. At our center, our policy has been to transect the pouch as it enters the pelvis and leave the remnant there. In this way, we avoid lengthy pelvic resections in an already complex operation.

This segment of bowel is inevitably disconnected from its vascular pedicle but only in two cases did this led to ischemic pouches that were treated conservatively with percutaneous drainage. One patient (patient 7) has had a delayed completion J-pouch removal via combined laparotomy and perineal approach.

### Retroperitoneal involvement

#### Ureteric involvement

**Fig. 2:**
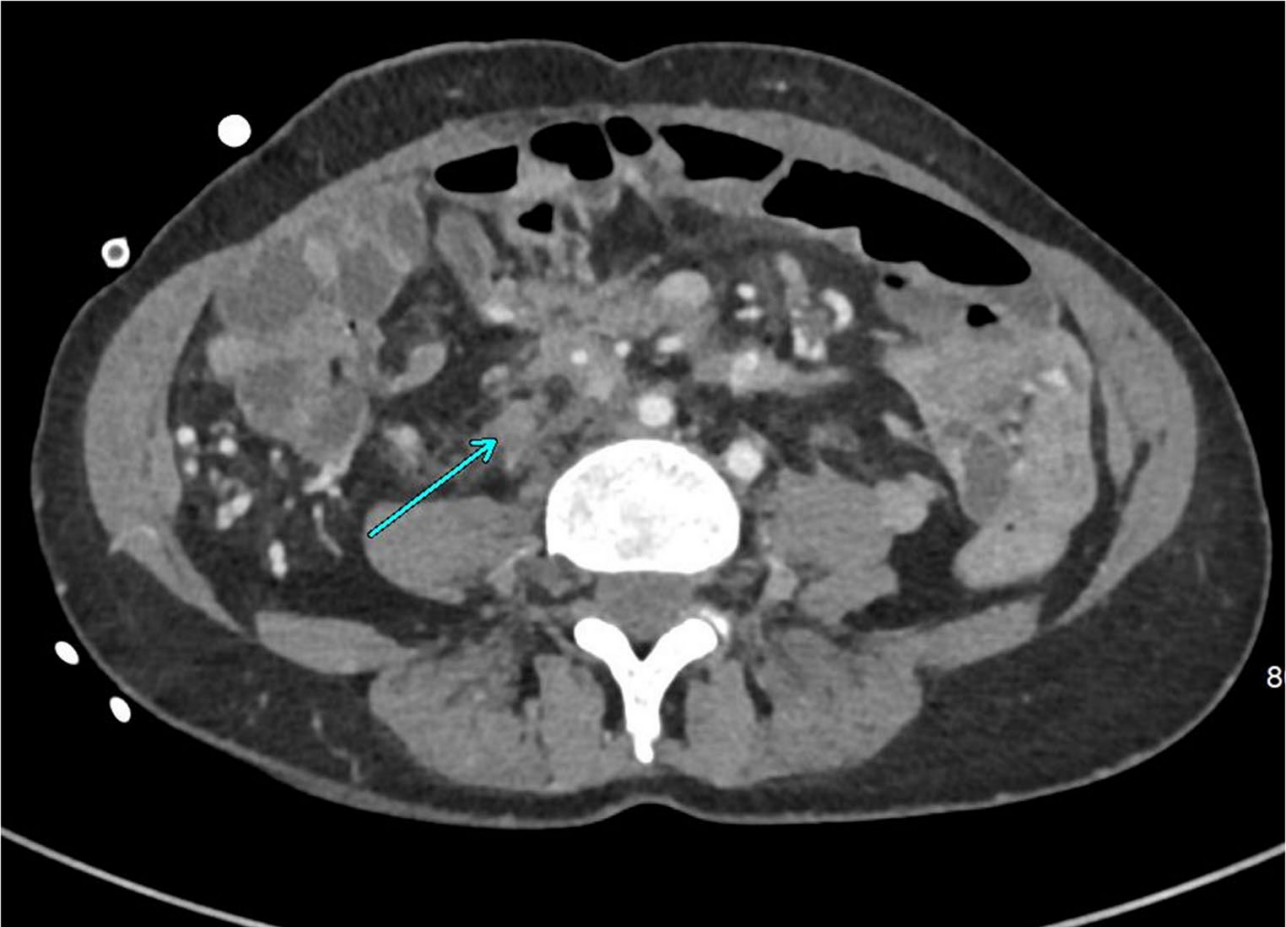
CT axial image, arrow indicates: right ureter encased with a desmoid tumour

Due to extensive nature of the desmoid disease, ureteric problems occurred frequently in our patients. In 8 patients, some level of significant ureteric involvement was present. Most frequently, we were confronted with unilateral involvement (*n* = 6, see Fig. 2). Two patients had bilateral ureteric involvement. In most cases, the ureter could be freed up and protected using stents. In one patient, a right-to-left uretero-ureteric anastomosis was performed, while three patients had to undergo renal auto-transplants due to extremely short remnant proximal ureters. Two patients had severe obstructive nephropathy with recurrent urosepsis and ultimately underwent native nephrectomies and allo-transplantation.

#### Vascular structures

**Fig. 3:**
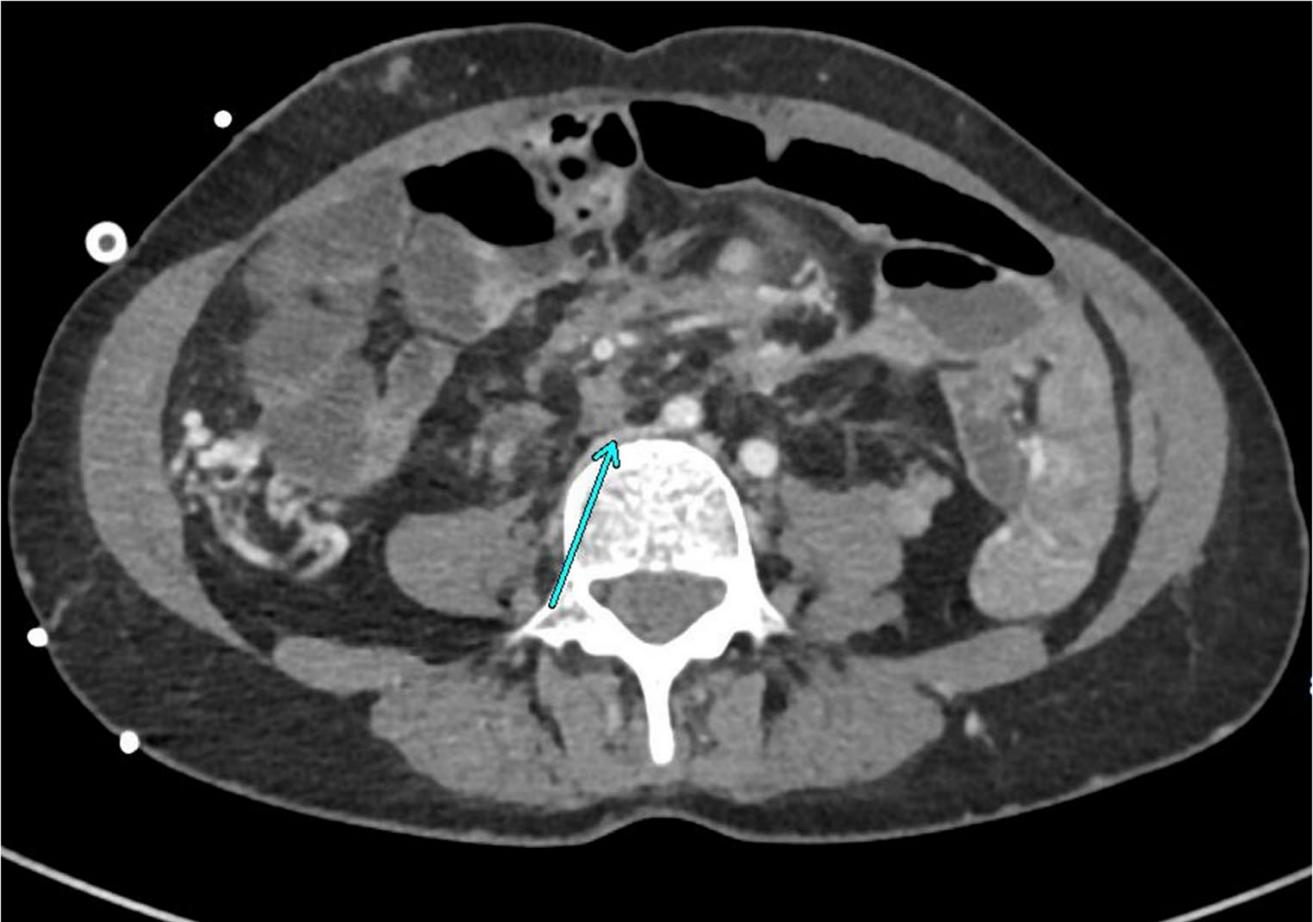
CT axial image: arrow indicates desmoid compressing iliac/inferior vena cava confluence

IVC compression by large desmoid masses was present in two cases. This required sharp dissection to free up and remove the tumor (see Fig. 3).

In another more extensive case (patient 1), the right iliac vessels (both common artery and vein) were encased by desmoid disease requiring a resection and reconstruction with donor vessels.

### Graft type selection

Our center aims to avoid inclusion of the liver in the ITx graft to both reduce the complexity of the operation and optimize the liver graft utilization for other patients. Despite extensive disease, the desmoid disease can be resected off the liver surface and thus only two patients required a liver (one for liver fibrosis and one for technical reasons).

Instead, the most frequently used grafts were either modified MVT (10 patients) or isolated ITx (8 patients). Due to extensive ureteric/renal involvement and associated renal disease, 2 patients also received a kidney allograft.

In those patients with limited and easily surveillable foregut adenoma’s – those with a low risk of malignant degeneration – we opted for the less complex isolated ITx. Conversely, if the risk was deemed too elevated, a foregut resection with modified MVT was chosen. Unfortunately, one patient did develop gastric adenocarcinoma – 6 years after transplantation - which ultimately proved fatal despite regular 6-montly endoscopic surveillance. At the time of listing, the endoscopy showed numerous tiny (< 3 mm) polyps in her duodenum (Spigleman score of 5) and a carpet of fundic polyps. Many biopsies were taken and were found to contain low grade dysplasia. As a result, the decision was taken to perform an isolated ITx and her foregut was monitored at 6-month intervals.

Due to the extensive pelvic disease and previous resections: all patients received a transplant colon as part of their graft which is brought out as an end-colostomy.

### Simultaneous versus sequential transplantation

Under ideal circumstances, a sequential resection – a resection followed by a delayed ITx – would be the most preferable by significantly shortening the length and the complexity of the operation. However, in most cases both resection and ITx had to be performed simultaneously (15 out of 20 patients). This was caused by the extent of the disease, which meant that a resection would leave the patient with a non-reconstructable GI tract or abdominal wall. By contrast, in the five sequential cases, the disease was more limited and thus the patients were transplanted primarily for short-gut related complications or desmoid recurrence after previous major resections.

### Desmoid recurrence

There were no instances of desmoid recurrence intra-abdominally, either within the graft or at the anastomosis with the native gut. However, due to FAP-caused genetic propensity for desmoid development, three patients did develop extra-abdominal tumors.

**Fig. 4:**
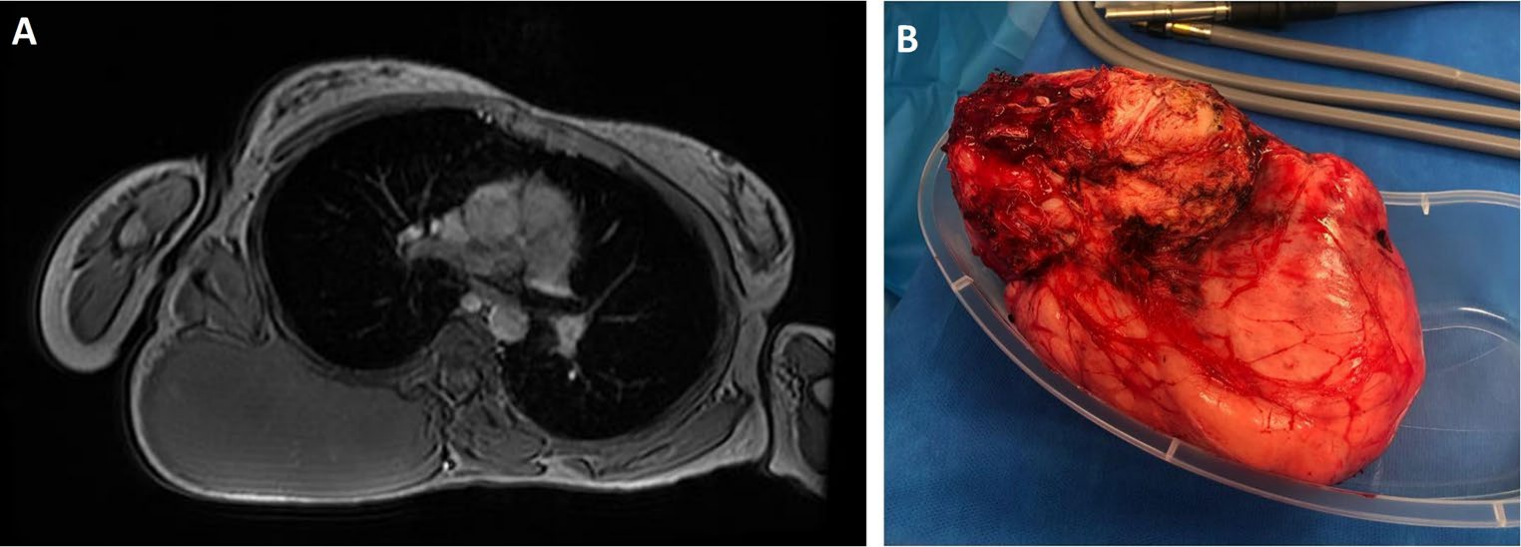
**A**: MRI axial image showing extra-abdominal desmoid - located in the thoracic paraspinal location. **B**: Photo of the desmoid once removed

Three patient required surgical resections for large extra-abdominal desmoid tumors, including one patient that had two operations for thoracic lesions (Fig. 4).

### Overall outcomes (Table 3)

The overall patient 5-year survival was 92% with a median follow up of 1575 days (60-4142). Two patients lost their graft – one was due to rejection and they since received a second graft. The second graft loss was due to post-transplant lymphoproliferative disorder induced intestinal perforation after initiating Rituximab treatment (130 days post-ITx).

Rejection occurred in 4 patients, most of whom were treated with pulse dose steroids. In the 5 patients that died since ITx, the majority was due to sepsis. One patient died from gastric cancer from her native stomach. The remaining 14 patients are alive with a functioning graft.

**Table 3:** Outcomes

Patient number	Patient survival (years)	Graft survival (years)	Re-transplantation	Rejection	Rejection treatment	Alive	Cause of death
1	8.7	0.6 8.7	Yes – second graft survival 8.7 years	No	N/A	No	Abdominal compartment syndrome – perforation and sepsis
2	5.3	5.3	No	Yes – Grade III (Severe)	Steroids /ATG	No	Chest sepsis
3	6.3	6.3	No	Yes – Grade I (Mild)	Steroids	No	Hyperkalemia induced cardiac arrest due to renal failure
4	11.4	11.4	No	NO	N/A	Yes	Alive
5	6.3	6.3	No	No	N/A	No	Metastatic gastric cancer
6	4.2	4.2	No	Yes – Grade II (Moderate)	Steroids	No	Urosepsis
7	6.9	6.9	No	Yes – Grade II (Moderate)	Steroids	Yes	Alive
8	6.5	6.5	No	No	N/A	Yes	Alive
9	6.2	6.2	No	No	N/A	Yes	Alive
10	5.0	5.0	No	No	N/A	Yes	Alive
11	4.4	4.4	No	No	N/A	Yes	Alive
12	4.2	4.2	No	No	N/A	Yes	Alive
13	4.2	4.2	No	No	N/A	Yes	Alive
14	2.5	2.5	No	No	N/A	Yes	Alive
15	2.2	2.2	No	No	N/A	Yes	Alive
16	0.9	1.9	No	No	N/A	Yes	Alive
17	1.6	1.6	No	No	N/A	Yes	Alive
18	1.2	1.2	No	No	N/A	Yes	Alive
19	0.7	0.4	No	No	N/A	Yes	Alive – lost graft and is back on PN
20	0.2	0.2	No	No	N/A	Yes	Alive

**Legend**: **ATG** = Anti-thymocyte globulin, **PN** = parenteral nutrition

## Discussion

In this study we present our updated figures for ITx for advanced FAP-related desmoid disease [11]. Our center has been performing transplantation for this indication since 2008 but there has been a significant increase in activity recently due to accrued experience leading to earlier referral from the main national FAP center (St. Mark’s in London, UK).

We found three main indications in our cohort:

1/Large, fistulating desmoid with difficult to control or recurrent abdominal sepsis.

2/Patient with recurrent desmoid disease after previous major intestinal resections that would lead to ultrashort gut syndrome.

3/Patients requiring foregut resections (gastric or duodenal resections) due to large adenomas at high risk for malignant degeneration but who lack sufficient remnant bowel length to complete a reconstruction.

The first category represents the largest group of patients which often have a very poor quality of life due. Furthermore, even though desmoids are considered benign and do not metastasize, they can lead to life-threatening complications. Large series have consistently demonstrated poor outcomes with up to 14–30% mortality [21, 22]. In these patients, ITx allows for maximal desmoid resection although the main aim is source control with removal of all the affected mesentery and associated collections.

Pelvic disease is frequently encountered and leads to specific surgical issues like ureteric, J-pouch or vascular involvement. Almost half of our patients presented with ureteric obstruction, which is a recognized complication in advanced desmoid disease [23]. Many patients presented with chronic ureteric obstruction with stents or even nephrostomies in situ. Often the distal ureters are drawn into the desmoid mass and therefore have to be meticulously resected off in order to spare them. Due to induration and encasement by the surrounding desmoid, this may not be possible and thus several of our patients have required either resections with reconstructions, diversions to the contralateral side (end-to-side anastomoses) or most radically an auto-transplantation. In this operation, first described in the context of FAP by Lattimer et al. [24], the affected kidney is removed with a short segment of desmoid free-ureter, flushed with preservation solution and subsequently implanted lower down in the pelvis (in the same location a regular allograft) on the iliac vessels. This will allow the kidney with the much shorter ureter, to be successfully reconnected to the bladder. This is important as maintaining renal function in ITx patients is vital, as these patients frequent have renal impairment post-operatively due to previous intestinal failure with chronic dehydration and immunosuppression burdens [19]. Some auto-transplants were difficult as pelvic disease made access to iliac vessels very difficult.

In the most extreme case, native nephrectomies had to be performed due to recurrent infections or due to anatomical difficulties to preserve the native kidney. One patient had a horseshoe kidney with numerous vessels and affected ureters. As such, an auto-transplant was not deemed feasible and thus a nephrectomy was combined with an allograft.

The ileo-anal J-pouch been gaining in popularity in prophylactic colectomies in FAP patients compared to the ileo-rectal anastomosis [25]. This has resulted in a higher proportion of patients presenting to us with pouches and complex desmoid disease in the pelvis. Furthermore, any pelvic surgery in FAP patients often stimulates new desmoid formation in this area [26]. As mentioned previously, when performing the resection at the time of ITx, we are constrained by an already lengthy procedure and limited preservation time of the intestinal graft. Therefore, we opt to resect the remanent intestine but leave the J-pouch in situ after disconnecting it from its mesentery. Two of our patients (out of 5) have developed pouch ischaemia that was treated conservatively with drainage. In theory, the best option would be a delayed removal of the pouch. However, only one patient in our series had this procedure so far due to the high risk for iatrogenic injury to nerve of vessels [27]. In our case, it required a combined laparotomy (to free up the loops of transplant small bowel) and perineal approach to remove the remnant pouch. While the pouch remains in situ, it should be regularly endoscopically surveilled due to the risk of pouch adenocarcinoma [28].

Significant vascular involvement of desmoid was relatively rare in our series. Two patients had significant compression of the distal inferior vena cava which made the resection difficult due to the lack of a clear surgical plane. One patient presented with extensive pelvic disease and encasement of the both common iliac artery and vein. This was reconstructed using the available donor iliac vessels at the time of transplantation.

Surgical resections in desmoid tumors are currently considered the option of last resort for patients due to the high rate of recurrence [29–31]. Interestingly, there is seem to be little effect of complete versus near-total resections in these patients [32]. With this in mind, the main focus of ITx is similar to non-transplant surgery for advanced desmoids: remove as much infection, desmoid-infiltration and obstructed/non-functional bowel. However, the availability of normal functional bowel in the graft does allow for much more aggressive debulking. Furthermore, these newly transplanted organs do not carry the underlying FAP genetic defects. This means that although intra-abdominal recurrence has been described after ITx [33], we did not see this in our cohort.

However, as in the non-transplanted population, FAP patients do frequently present with large, extra-abdominal desmoids [34]. In our series, 3 patients had recurrences in the abdominal wall (native) and the thoracic area which did require surgery.

In an effort to reduce the recurrence rate and slow the growth of remnant desmoid disease, our team has recently been placing our FAP patients specifically on sirolimus (rapamycin) which is a mammalian target of rapamycin inhibitor (mTOR). This was initially done in many of our ITx patients to protect the renal function regardless of indication. However, based on some limited data on its anti-proliferative effect in desmoids, we now routinely start sirolimus after 3–6 months post-transplant [35].

Another contentious point, is the extent of native organ resection and subsequent choice of graft. Essentially, there are two options: foregut-sparing isolated ITx or foregut resection with modified MVT. The former is a shorter, less complex surgery with a lower complication rate and quicker recovery. By contrast, modified MVT is a much more complex operation with worse short-term outcomes. While initially leaning more towards the limited transplantation, the death by gastric cancer in patient 5, did lead us to now have a lower threshold for modified MVT. This applies especially to those patients with extensive ‘carpeting’ disease of the stomach or numerous duodenal polyps. This is especially driven by the increasing incidence of gastric and duodenal cancer, which have a very poor prognosis [36–38]. Furthermore, one could imagine that chronic immunosuppression after ITx would further increase this risk.

Similar to the retroperitoneal organs, the abdominal wall is often affected either directly by fistulation or direct invasion [39]. Furthermore, due to extensive resections, the abdominal domain may be severely restricted. Thus abdominal closure can be a major challenge. While various methods have been used in the past, we now exclusively rely on non-vascularized rectus fascia procured from the same donor as the bowel to close the abdomen [40, 41]. This simple, inexpensive and reliable method allows for bridging and closure of significant defects while allowing sufficient space for the new graft.

Finally, given the immense complexity of both the explant and the subsequent implant, the operation should ideally be split into two distinct stages. However, due to expected longer waiting time on the list and unpredictable graft availability, an extensive exenteration and subsequent listing is a potential but risky strategy. In our more recent experience, due to the extent of the disease, most patients have required extensive resections that necessitate simultaneous operations to reconstruct the GI tracts. Ideally, multiple teams that can rotate and take appropriate rests are warranted for these very long and complex operation (some exceeding 20 h).

Despite this study providing valuable insight into this rare disease, we have to acknowledge some limitations. First, due to the retrospective nature, some data may not have been included or lost. However, it should be noted that no patients were lost to follow-up. Second, there is a likely to be significant referral bias as only those patients considered eligible for ITx (relatively young patients, with no other known comorbidities) would be considered for this pathway. We do not have exact figures on how many patients were considered but eventually declined referral. However, given the UK’s existing strongly centralized pathway to identify and treat hereditary colorectal cancer/polyposis disease, this data could help create more evidence based referral guidelines. As a result of the strict referral criteria and rare nature of this disease, the absolute number of patients undergoing ITx remain very limited.

Finally, we were not able to report any patient-reported outcome measures (PROMs) as we did not prospectively capture these. However, last year a validated, disease-specific questionnaire has been developed which we could use for future research endeavors [42].

## Conclusion

Intestinal transplantation (ITx) is a viable option for specific patients with severe desmoid disease when all other treatment options have been exhausted. It remains crucial to determine which patients would benefit from ITx to ensure timely referrals. Despite high morbidity, outcomes are excellent in experienced hands with survival exceeding 90% in patients that would otherwise have very limited options.

## Data Availability

No datasets were generated or analysed during the current study.

## Abbreviations

FAP: Familial Adenomatous Polyposis
ITx: Intestinal Transplantation
IVC: Inferior Vena Cava
MVT: Multivisceral Transplantation
